# Parturient Apoplexy under Schmidt’s Treatment

**Published:** 1900-05

**Authors:** J. H. Tennent

**Affiliations:** London, Ont.


### REPORTS OF CASES.

### PARTURIENT APOPLEXY UNDER SCHMIDT'S TREATMENT.

By J. H. Tennent, V.S.,
LONDON, ONT.

The same instructions as to care and the same antiseptic precautions were used in each and every case unless otherwise specified.

Schmidt's treatment and our mode of applying it are as follows : Procure a clean vessel (a quart fruit sealer will do, and we prefer something of this kind, as it can be kept closed until the udder is prepared to receive it). Put into the vessel a quart of boiling water, and when cooled to a temperature of 100° or 101° F., add formalin, ʒj, and potassium iodide, ʒij ; shake gently, and it is ready for use.

Remove all the milk possible from the udder, then wash the udder and teats with an antiseptic wash ; also the instrument, which consists of an ordinary bulb enema syringe (human), to which is attached a teat siphon—a jeweller brazing a shoulder on to a siphon so as to fit the end of the syringe. The siphon is passed up the milk duct of the teat, and eight ounces of the solution of potassium iodide-formalin and water is injected into each quarter of the udder. In emptying the contents of the syringe into the gland air is admitted. I have seen no bad results, but would not advise too much air to be forced in, as it might be the means of infection of other germs.

If the patient is down and unable to rise, a clean cloth should be placed under the udder to keep it out of the stable litter.

*Care.* Place the animal in a position on the sternum, and endeavor to keep her so by bundles of straw, etc. ; clothe the body according to the season ; turn her from side to side every five or six hours. The udder is to be hand-rubbed or kneaded every hour, so as to ensure distribution throughout the entire gland. The solution is not to be milked out of the udder before eight or ten hours, when a second injection may be given if required.

Remove feces from the rectum. If urine is retained over twelve hours use the catheter. And in no case must medicines be administered by the mouth until the patient is well able to swallow. We give no medicine by the mouth unless the patient is able to stand.

Cold water may be given in small quantities frequently if the patient will drink. Food during convalescence consists of something light, nourishing, and easily digested. If constipation is present two or three days after the commencement of an attack an ordinary physic drench may be given.

The other medicinal treatment will be given with each case.

CASE I. *February* 28, 1899. Saw cow at 9.30 P.M. Down, unable to rise. Eyes amaurotic and all other symptoms well marked; pulse 96 and strong; temperature 101° F. Calved thirty-six hours previous. Extracted six quarts of blood from the jugular with difficulty. Gave aloes barb. ℥j, sod. chlor. ½ lb., spts. eth. nit. ℥ij, water 1 qt. Injected udder. Left eth. nit. to be given in ℥ij doses every five hours. Usual directions as to care, etc.

*March* 1*st*, 10 A.M. Still down; symptoms much better; bowels moving freely; urine high colored; pulse 98 and weaker; temperature 100° F. Two quarts from udder. Respirations normal. Injected udder. Left eth. nit., ℥ij, to be given as before, and same care, etc. 8 P.M. Had been up twice during the afternoon; had eaten a little and drank some water; found her ruminating slowly. Pulse very fast and weak. Temperature 102° F. Partial sweats on body; eyes discharging a watery fluid. Ordered three ounces of whiskey and left nux vomica to be given in ℥j doses, with the whiskey, every four hours.

2*d*, 10 A.M. Cow up and doing well, eating and drinking; pulse strong, 60; temperature 100.5° F.; not much milk from udder. Left pulv. nux vom., sod. bicarb., ginger, and gentian, ℥j each, to be given with one ounce of whiskey three times a day. Three days after the owner reported the cow doing well, but not as large a flow of milk as he expected.

CASE II. *March* 7*th*, 7 P.M. Patient down, but got up with difficulty. Paddling for a few moments, then went down again. Very fat; body covered with perspiration; respiration fast and difficult; pulse fast and weak; temperature 103.6° F.; bowels moved but little, and constipated since calving; voided a large amount of clear urine; calved twenty-four hours previous. Injected udder. Ordered ice to the head, left usual directions, but no medicine.

8*th*, 8.30 A.M. Patient down but got up and stood steady for a short time. Respirations stertorous; temperature 101° F.; pulse imperceptible; all other symptoms favorable. Injected udder and ordered ℥iij whiskey every three hours; same care, etc. 6.30 P.M. Gets up and down often. Respiration labored; pulse imperceptible; temperature 101° F.; bowels moving and urine voided.

Left pulv. nux vom., ʒjss ; whiskey, ℥ij, to be given every three hours ; same care, etc.

*9th*, 9 A.M. No perceptible change, and left same medicine as before. 5.30 P.M. Bowels moving ; urine voided ; drank one-half pail of water ; ate small bran mash ; pulse stronger, but still weak ; temperature 100° F. Prognosis favorable. Gave ol. lini, 1 pint ; ol. tereb., ℥j, and left same medicine except the whiskey, it to be given only one-half the quantity every four hours.

*10th*. Up and doing well. Left powder of sod. bicarb., ginger, and gentian, ʒj each, to be given three times a day.

CASE III. *April 4th*, 7.30 A.M. Jersey cow. Down and unable to rise. Symptoms well marked ; saliva flowing freely ; temperature 101.6° F. ; pulse 98 and strong ; cow in high condition and a very rich milker ; calved fourteen hours previous. Injected udder as usual. Left eth. nit., ℥ij doses, every four hours if patient was able to swallow. 7 P.M. Not any perceptible change ; breathing fast and difficult ; gave enema and left nux vom., ʒj doses, and rum, ℥iij, every four hours. Injected udder.

*5th*, 9 A.M. Still down ; head up ; temperature 103° F. ; pulse 80 and firm ; bowels moving freely ; ate bran mash and clover hay and drank water ; same treatment as night before. Injected udder again. 5 P.M. Still down, but all symptoms favorable ; did not inject udder ; gave same doses as before.

*6th*, 3 P.M. Still down ; temperature 100.2° F. ; pulse 76 and strong ; symptoms favorable ; left whiskey, ℥ij doses every six hours.

*7th*, 8 P.M. Up and doing nicely and made a good recovery.

CASE IV. Cow, very fat and heavy milker ; calved twenty-eight hours previous ; pulse 96 ; temperature 101° F.; paddling of hind legs ; went down while there, but got up again. Gave chlor. sod., 1 lb.; aloes barb., ʒjss ; liq. am. acet., ℥iij. Injected udder and left nux vom., ʒj dose to be given every four hours. Next day owner reported cow doing well.

CASE V. *May 31st*, 9 P.M. Pure bred Ayrshire, very fat and heavy milker ; calved twenty-eight hours previous ; down ; delirious ; pulse imperceptible ; temperature 102° F. ; cold sweats on body ; respirations difficult ; could not swallow a particle. Injected udder only ; usual care.

*June 1st*, 8 AM. Cow standing ten minutes when I got there ; bowels moving freely ; pulse 80 and firm ; temperature 100° F. ; respirations natural. Injected udder and prescribed a pint of ale and one drachm of nux vomica every four hours.

2*d*. Cow doing well; out grazing on the lawn, and made a rapid recovery.

Case VI. *June 8th*, 11.30 A.M. Large cow, very fat and heavy milker, was down and comatose. Pulse imperceptible; temperature 97° F.; breathing fast and difficult; water running from eyes and saliva from mouth. Tympanites present. Injected udder only; strict orders as to care. 9 P.M. Cow up; drank some water; bowels moving; symptoms favorable. Left liq. acet. am., ℥ij doses every six hours. Injected udder. Owner reported cow all right two days afterward.

Case VII. *June 10th*, 7.30 A.M. Had calved forty-eight hours previous; placenta had been removed thirty-six hours after calving, when one pound of mag. sulph. was given. Fat, and medium milker; down, unable to rise; pulse 92; temperature 100.2° F.; respirations natural. Injected udder, and left liq. am. acet., ℥ij to be given eight hours afterward. 6.30 P.M. Still down; symptoms favorable. Injected udder, and left liq. am. acet., ℥ij doses to be given every six hours.

11*th*. Owner reported cow up and doing well. Gave six powders of sod. bicarb., gentian, ginger, and nux vom., ʒj each, a powder to be given three times a day.

Case VIII. *June 10th*, 8 A.M. Calved forty hours; very fat and a heavy milker; delirious; down; pulse weak and fast; temperature 102° F.; breathing fast and sonorous. Injected udder and left usual directions. 11.30 A.M. No change in symptoms; only able to swallow, and gave ℥iij of whiskey. 6 P.M. Cow up; drank some water; symptoms very favorable. Injected udder and ordered whiskey to be given, to which owner objected, saying he had a pain killer of his own manufacture that cured all the ills of man and animal, to which we strongly objected, but he gave her teacupful doses every two hours, and in twenty-four hours the cow was dead.

Case IX. *June 13th*, 7.30 A.M. Calved forty-eight hours previous; placenta was removed thirty-six hours after calving, when one pound mag. sulph. was given. Down; fat; fair milker; comatose; injected udder; usual directions; left no medicine. 6.30 P.M. Cow up; symptoms favorable; injected udder, etc.; left whiskey, ℥ij doses every six hours.

14*th*. Owner reported cow doing well; gave powders same as Case VII.

Case X. *June 15th*, 8 A.M. Fat, heavy milking Holstein; was down, but got up with difficulty; paddled with hind legs, and fell again; head to one side and all the well-marked symptoms of the disease. The owner informed me that she had not yet calved, but he had been milking her for four days, as her udder was of a tremendous size, and milk was oozing out of the teats. Examined her per vagina, and found feet and nose in passage and fœtal membranes intact; broke them, and they seemed very tough, and with a little help she was delivered of a big, healthy calf. She was lying under a tree about two miles from the stable, and it rained for a couple of hours very hard. Injected udder; left usual directions; ordered body to be rubbed dry and blanketed and made as comfortable as circumstances would permit. Left liq. acet. am., ʒij, to be given seven hours afterward. 6 P.M. Cow still down; head up; bowels moving freely; voided urine; pulse and temperature almost normal; drank one-half pail of water. Injected udder. Left liq. am. acet. and whiskey, each ʒij, to be given every five hours. Afterbirth came away three hours after delivery of calf.

16*th*. Owner reported that the cow got up about four hours after the last injection, and during the night walked over to the stable, where he left her ruminatng quite contentedly; gave same powders as Cases I. and VII.

Case XI. *June* 23*d*, 6 A.M. Calved twelve hours previous; fat and a heavy milker; down and unable to rise; weather very hot; placed her on a stone boat and brought to the stable. Respiration difficult; pulse weak and fast; temperature 97° F. Injected udder and left usual directions.

23*d*, 5 P.M. Cow up and all symptoms relieved; removed placenta and injected uterus with creolin solution; gave mag. sulph., 1 pound; aloes barb., ʒij; whiskey, ʒij. Injected udder. Left whiskey, ʒij, nux vom., ʒj, to be given every six hours.

24*th*. Owner reported cow doing nicely; gave powders same as Cases I. and VII.

Case XII. *July* 12*th*, 10 A.M. Calved twenty hours previous; fair condition, but a very heavy milker; down and unable to rise; comatose; bowels tympanitic; respirations labored; pulse imperceptible; temperature normal. Injected udder; left usual directions. 6 P.M. Up and doing well; symptoms favorable. Injected udder; left tr. nux vom., ʒj, and whiskey, ʒiij, to be given every three hours. Next morning owner reported that he had removed the placenta, and it was very lumpy, and that there was profuse hemorrhage from the vagina, and the cow died in a few hours. There was no evidence that the placenta was retained when I saw her, and the owner informed me that she had cleaned, he evidently mistaking the membrane for the placenta.

CASE XIII. Cow calved fourteen hours previous; very fat and medium milker. Down, but got up with difficulty; symptoms well marked. Injected udder; usual care, etc. Saw her the next morning, and she was apparently well. Left powders the same as Case VII. Owner reported in a couple of days that the cow was all right.

CASE XIV. *October 9th*, 8 A.M. Ayrshire cow. Calved forty-eight hours previous; in good flesh; a very heavy milker. Down and unable to rise; pulse weak and fast; temperature 101° F. Injected udder; usual directions. 4 P.M. Still down, ruminating slowly; pulse and temperature improving; appetite returning; voided urine; bowels moving. Injected udder; same care; left liq. am. acet., spts. eth. nit., and whiskey, ℥j each, to be given every five hours until three doses were given Owner reported the next morning that the cow was up and apparently well, except slight constipation. Gave mag. sulph., 1 pound; aloes barb., ℥jss; same powders as Case VII., with one-half ounce of sod. hyposulph. added. Cow made a complete recovery.

CASE XV. *November 13th*, 11 A.M. Holstein cow, fat, and very rich milker; calved twenty-four hours; down and completely helpless; pulse imperceptible; temperature 1° F. below normal. Injected udder and instructions as to care; nothing to be given by mouth. 8 P.M. Cow up; symptoms much better; injected udder; same care. Left liq. am. acet., ℥ij doses, every five hours.

14*th*, 9 A.M. Cow up and doing well. Left powders same as Case VII. In three days owner reported bowels constipated. Gave mag. sulph., 1 pound; aloes barb., ℥jss. The cow made a complete recovery.

The above is a correct report of all the cases of parturient apoplexy treated by us during 1899. Of the fifteen cases treated, thirteen cows are alive, and I am of the opinion that if the owners had not interfered the other two would have recovered. Case I. did not give as large a flow of milk this season as usual. In Case VI. the hair all fell off of the tail. In Case VII. one-quarter of the udder was badly swollen for a few weeks, but came all right. You will notice that Case V. and all cases since received very little medicine. They were our worst cases, and made the speediest recoveries. Cases previous to Case V. were not so severe, and were tardy in their recovery, due, I think, to pouring down too much medicine. In the future we will give no more medicine than the case actually requires, and then not until the patient is able to stand or well able to swallow. The greatest difficulty with us in severe cases is to keep up the heart's action, and have had best results from nux vomica and whiskey, given in doses and frequency according to the case. Pardon me for trespassing on your time and patience with so lengthy an article. If you deem any of it worthy of publication you are at liberty to use it. Any correspondence in connection with this or any other case will be cheerfully answered.

